# Therapeutic Failure and Acquired Bedaquiline and Delamanid Resistance in Treatment of Drug-Resistant TB

**DOI:** 10.3201/eid2905.221716

**Published:** 2023-05

**Authors:** James Millard, Stephanie Rimmer, Camus Nimmo, Max O’Donnell

**Affiliations:** Guys and St Thomas' NHS Foundation Trust, London, UK (J. Millard);; Wellcome Trust Liverpool Glasgow Centre for Global Health Research, Liverpool, UK (J. Millard);; University of Liverpool, Liverpool (J. Millard);; Africa Health Research Institute, Durban, South Africa (J. Millard, C. Nimmo);; Imperial College Healthcare, London (S. Rimmer);; Francis Crick Institute, London (C. Nimmo);; University College London, London (C. Nimmo);; Columbia University Medical Center, New York, New York, USA (M. O’Donnell);; CAPRISA MRC-HIV-TB Pathogenesis and Treatment Research Unit, Durban (M. O’Donnell)

**Keywords:** tuberculosis and other mycobacteria, bacteria, antimicrobial resistance, bedaquiline, delamanid, XDR TB, South Africa

## Abstract

New classes of antitubercular drugs, diarylquinolines and nitroimidazoles, have been associated with improved outcomes in the treatment of drug-resistant tuberculosis, but that success is threatened by emerging drug resistance. We report a case of bedaquiline and delamanid resistance in a 55-year-old woman in South Africa with extensively drug-resistant tuberculosis and known HIV.

Major improvements have been achieved in drug-resistant tuberculosis (TB) treatment in recent years; 2 new drug classes, diarylquinolines (bedaquilline) and nitroimidazoles (pretomanid and delamanid), have been central to this success (*1*). The ZeNiX trial demonstrated cure rates of >90% in complex drug-resistant TB when a bedaquiline, pretomanid, and linezolid (BPaL) regimen was given for just 6 months (*2*); the TB-PRACTECAL trial used BPaL plus moxifloxacin to treat multidrug-resistant (MDR) TB and achieved cure rates of 89% versus 50% in controls (*3*). These regimens have been recommended as the preferred treatment option for drug-resistant TB by the World Health Organization (*4*).

Threatening those recent successes, resistance can emerge to bedaquiline through mutations in *atpE*, *pepQ*, or *Rv0678* (with mutations in *Rv0678* being by far the most common in clinical isolates) and to pretomanid and delamanid through mutations of *fbiA/B/C*, *ddn*, and *fgd1*. Bedaquiline resistance has been increasingly reported, but only a handful of cases of resistance to both classes of agents in TB patients have been reported (*4**–**8*). All of those reports have described prolonged infections treated with differing drug combinations over time, and none were cases where all 3 components of BPaL were administered concurrently. We present the case of a patient with pre-XDR TB (resistant to rifampin, isoniazid, and fluoroquinolones) in South Africa whose treatment regimen failed when bedaquiline resistance and subsequent delamanid resistance developed, despite treatment with bedaquiline, delamanid, and linezolid beginning early in therapy.

A 55-year-old woman sought care in July 2018 for weight loss and fatigue. She had completed treatment for drug-susceptible TB in 2007 and received an HIV diagnosis in 2012 (initial viral load 31,222 copies/mL and CD4 count 174 cells/μL) that was treated with efavirenz/emtricitabine/tenofovir. Chest radiography revealed extensive cavitary pulmonary TB. She had no other medical diagnoses. Initial sputum smear showed 3+ acid-fast bacilli and was culture-positive for *Mycobacterium tuberculosis* consistent with rifampin resistance by the Xpert MTB/RIF assay (Cepheid, https://www.cepheid.com).

A 9-month oral regimen consisting of bedaquiline, linezolid, clofazimine, levofloxacin, ethionamide, and pyrazinamide was commenced in line with guidelines in South Africa at the time, and the patient was enrolled in the PRAXIS study, a randomized controlled trial of bedaquiline adherence support (*9*). Antiretroviral drugs were changed to nevirapine/emtricitabine/tenofovir to manage interactions with bedaquiline. GenoType MTBDRplus and MTBDRsl (Hain Lifescience, https://www.hain-lifescience.de) line probe assay results and phenotypic drug susceptibility testing (DST) on the baseline culture confirmed resistance to rifampin, isoniazid, and fluoroquinolones. Because of fluoroquinolone resistance, we changed treatment to a modified World Health Organization long regimen (minimum 18 months) and added terizidone and p-aminosalicylic acid. Delamanid became available through an expanded-access program and was added at week 6 when levofloxacin and p-aminosalicylic acid were stopped.

We assessed adherence to bedaquiline and antiretrovirals using a Wisepill medication dispenser (Wisepill Technologies, https://www.wisepill.com). We conducted extended-phenotypic DST at weeks 6 and 34 and performed whole-genome sequencing (WGS) at weeks 6, 14, and 34. We assessed MICs for a range of drugs on week 6 isolates and week 34 isolates by using a microtiter plate (*9*). Phenotypic DST at week 6 demonstrated additional resistance to moxifloxacin, ethionamide, ethambutol, streptomycin, and kanamycin; we observed corresponding mutations in *rpoB*, *katG*, *gyrA*, *ethA*, *embB*, and rrs (Figure; Appendix Figure). At week 10, the patient was discharged from hospital for outpatient management.

**Figure:**
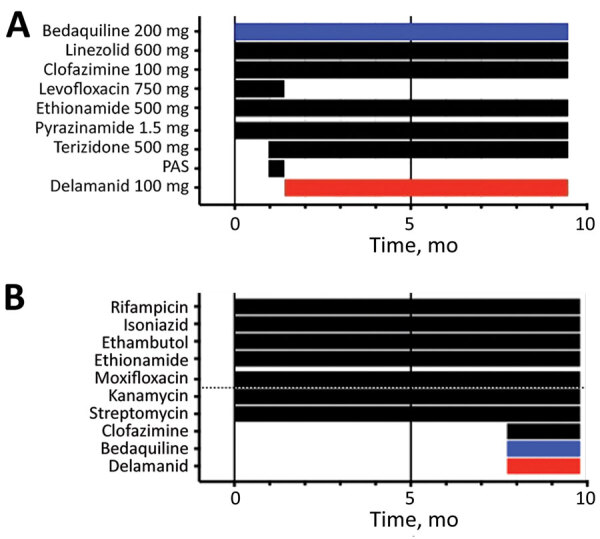
Drug regime over time of patient with drug-resistant tuberculosis, South Africa. A) Drug treatment history by month. B) Development of drug resistance according to phenotypic drug susceptibility testing per month. Red indicates delamanid, blue indicates bedaquiline. PAS, P-aminosalicylic acid.

Despite therapy, she remained smear-positive and culture-positive at week 34 and had lost a further 5 kg. We extended bedaquiline to 9 months. Bedaquiline adherence from initiation of therapy was 45%. WGS at week 14 identified acquisition of 2 bedaquiline resistance-associated variants (RAVs) in *Rv0678*: one was a single-nucleotide variation (Gln22Pro) and the other was an insertion (Asp47frameshift). WGS at week 34 demonstrated acquisition of a further 6 *Rv0678* bedaquiline RAVs (Ala57Glu, Arg72Trp, Asp88frameshift, Asp88Ala, Gly121Arg, Leu122Pro) and the emergence of 2 heterozygous *fbiC* loss-of-function mutations (Ala487frameshift, 25%; Ser534stop, 12%). Phenotypic DST at week 34 confirmed emergent resistance to bedaquiline and clofazimine, which commonly demonstrates cross-resistance with bedaquiline because of *Rv0678* mutations (Figure). Delamanid phenotypic DST by microtiter plate confirmed an increase in delamanid MIC from 0.015 to >0.5 µg/mL, consistent with resistance (*10*). The patient died ≈10 months after initial diagnosis and treatment initiation.

Recent clinical trial evidence from ZeNix-TB and TB-PRACTECAL has been extremely encouraging for the development of an effective, 6-month regimen for complex drug-resistant TB (2,3). Despite promising clinical trial results, this case highlights the ease with which resistance can develop in real-world implementation, likely because of the complex interplay of factors such as inadequate regimens caused by delayed or limited DST, drug pharmacokinetics, lesion penetration of drugs, and medication adherence. As regimens based on BPaL are rolled out more widely, combining this treatment with contemporaneous access to rapid DST for all agents, and access to adherence support, is essential to limit the development of resistance and loss of these effective new regimens.

Appendix Additional information about therapeutic failure and acquired bedaquiline and delamanid resistance in treatment of drug-resistant tuberculosis.
