# Supplementary material for: Therapeutic Failure and Acquired Bedaquiline and Delamanid Resistance in Treatment of Drug-Resistant TB
Source: Emerg Infect Dis. 2023 May;29(5):1081–4. doi: 10.3201/eid2905.221716 (PMC10124645; doi:10.3201/eid2905.221716)
Supplement: Appendix — Additional information about therapeutic failure and acquired bedaquiline and delamanid resistance in treatment of drug-resistant tuberculosis. [file 22-1716-Techapp-s1.pdf]

Article DOI: http://doi.org/10.3201/eid2905.221716

EID cannot ensure accessibility for Supplemental Materials supplied by authors. Readers who have difficulty accessing supplementary content should contact the authors for assistance.

Therapeutic Failure and Acquired Bedaquiline and Delamanid Resistance in Treatment of Drug-Resistant TB

Appendix

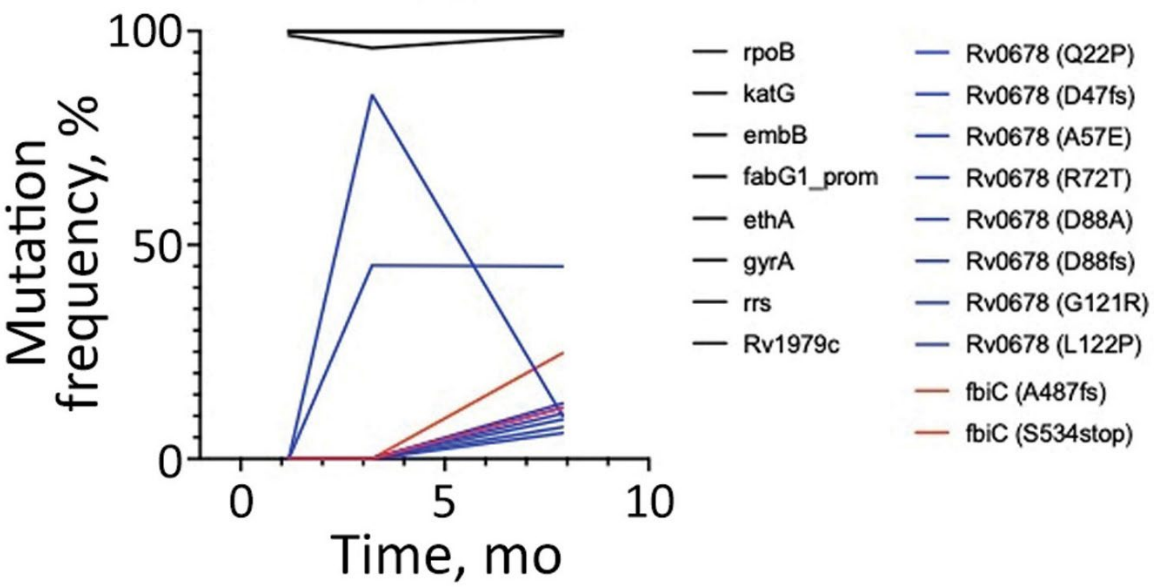

Appendix Figure. Whole-genome sequencing mutations and their frequencies over time color matched to corresponding development of drug resistance over time. Red indicates delamanid resistance, blue indicates bedaquiline resistance.
